# Benchmark White Matter Hyperintensity Segmentation Methods Fail on Heterogeneous Clinical MRI: A New Dataset and Deep Learning–Based Solutions

**DOI:** 10.1007/s10278-025-01808-9

**Published:** 2026-01-29

**Authors:** Junjie Wu, Joshua D. Brown, Ranliang Hu, Paula J. Edwards, Allan I. Levey, James J. Lah, Deqiang Qiu

**Affiliations:** 1 Department of Neurology, School of Medicine, Emory University, 1364 Clifton Rd NE, Atlanta, GA 30322 USA; 2 Department of Radiology and Imaging Sciences, School of Medicine, Emory University, 1364 Clifton Rd NE, Atlanta, GA 30322 USA; 3 Department of Biostatistics and Bioinformatics, Rollins School of Public Health, Emory University, Atlanta, GA USA; 4 Goizueta Alzheimer’s Disease Research Center, Emory University, Atlanta, GA USA; 5 Joint Department of Biomedical Engineering, Emory University and Georgia Institute of Technology, Atlanta, GA USA

**Keywords:** White matter hyperintensities, Clinical MRI, Deep learning segmentation, nnU-Net, Foundation models

## Abstract

Existing automated methods for white matter hyperintensity (WMH) segmentation often generalize poorly to heterogeneous clinical MRI due to variability in scanner types, field strengths, and protocols. To address this challenge, we introduce a diverse clinical WMH dataset and evaluate two deep learning–based solutions: an nnU-Net model trained directly on the data and a foundation model adapted through fine-tuning. This retrospective study included 195 routine brain MRI scans acquired from 71 scanners between June 2006 and October 2022. Participants ranged in age from 46 to 87 years (median, 70 years; 94 females). WMHs were manually annotated by an experienced rater and reviewed under neuroradiologist supervision. Several benchmark segmentation methods were evaluated against these annotations. We then developed Robust-WMH-UNet by training nnU-Net on the dataset and Robust-WMH-SAM by fine-tuning MedSAM, a vision foundation model. Benchmark methods demonstrated poor generalization, frequently missing small lesions and producing false positives in anatomically complex regions such as the septum pellucidum. Robust-WMH-UNet achieved superior accuracy (median Dice similarity coefficient [DSC], 0.768) with improved specificity, while Robust-WMH-SAM attained competitive performance (median DSC up to 0.750) after only limited training, reaching acceptable accuracy within a single epoch. This new clinically representative dataset provides a strong foundation for developing robust WMH algorithms, enabling fair cross-method comparisons, and supporting the translation of segmentation models into routine clinical practice.

**Supplementary Information:**

The online version contains supplementary material available at https://doi.org/10.1007/s10278-025-01808-9.

## Introduction

White matter hyperintensities (WMHs) are a common imaging marker of cerebral small vessel disease, especially in older adults and in patients with cerebrovascular or neurodegenerative conditions [1]. Their presence and volume are associated with cognitive impairment, dementia, and elevated risk of stroke [2]. Consequently, accurate quantification of WMHs is of great clinical and research importance. However, manual lesion annotation, the clinical gold standard, is extremely labor-intensive and subject to substantial intra- and inter-rater variability [3], motivating the development of automated segmentation methods.

Over the past decade, a wide variety of WMH segmentation techniques have been proposed [4, 5]. Early approaches based on intensity thresholding, region growing, or probabilistic modeling have gradually been supplanted by modern machine learning methods. Among traditional methods, the Lesion Segmentation Tool (LST) remains widely used, as its algorithms have long been regarded as high-performing solutions for WMH segmentation [4]. More recent methods, including LST-AI [6] and WMH-SynthSeg [7], seek to further improve accuracy and robustness. In particular, convolutional neural networks based on the U-Net architecture have achieved state-of-the-art results on benchmark datasets such as those used in the WMH Segmentation Challenge 2017 [8].

However, the majority of these models have been developed and validated on standardized research datasets acquired under controlled imaging protocols. In contrast, clinical MRI scans exhibit considerable variability in resolution and artifact levels due to differences in scanner models, field strengths, acquisition settings, and patient characteristics [9]. Such heterogeneity poses a major challenge for generalization, i.e., models trained on clean research images often fail to maintain performance when applied to routine clinical images [3].

To address this limitation, we curated a new dataset of clinical MRI scans with expert WMH annotations, specifically designed to capture the variability and imperfections commonly encountered in real-world clinical imaging. We evaluated several widely used and state-of-the-art WMH segmentation methods on this dataset. Despite their high performance on research benchmarks, these methods failed to produce reliable WMH segmentations on our clinical images, revealing a substantial gap in their robustness and generalizability. The dataset is available at https://doi.org/10.5281/zenodo.18339994.

To bridge this gap, we trained a deep learning–based segmentation model using the nnU-Net framework, a self-configuring pipeline that automatically optimizes its architecture, preprocessing, and training regimen based on the characteristics of the input data [10]. When evaluated on our challenging clinical dataset, the nnU-Net model demonstrated markedly improved performance compared to existing methods, achieving close concordance with expert annotations. In parallel, we investigated whether foundation models could enhance generalizability to real-world clinical settings. Specifically, we fine-tuned MedSAM, a segment-anything transformer pretrained on a large and diverse corpus of medical imaging data [11], to assess its capacity to transfer broad segmentation knowledge to the task of WMH delineation. This dual strategy enabled a systematic evaluation of both task-specific optimization and cross-domain knowledge transfer as potential solutions to the generalization challenges inherent in clinical WMH segmentation.

## Materials and Methods

### Data Acquisition and Annotation

**Fig. 1:**
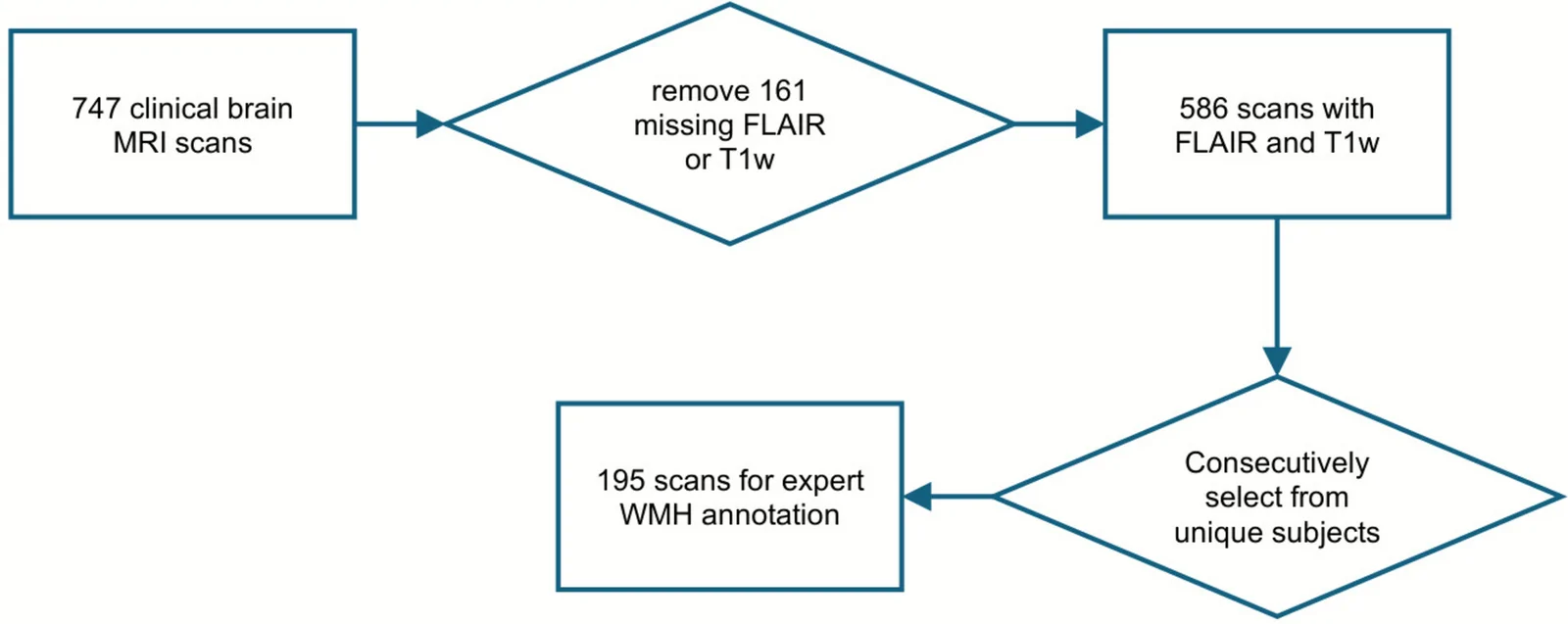
Flow of MRI scan selection for WMH annotation. Of 747 routine clinical brain MRI scans, 586 scans included both T1-weighted and axial T2-weighted FLAIR sequences. From these, 195 scans from unique participants were selected for expert WMH annotation

**Fig. 2:**
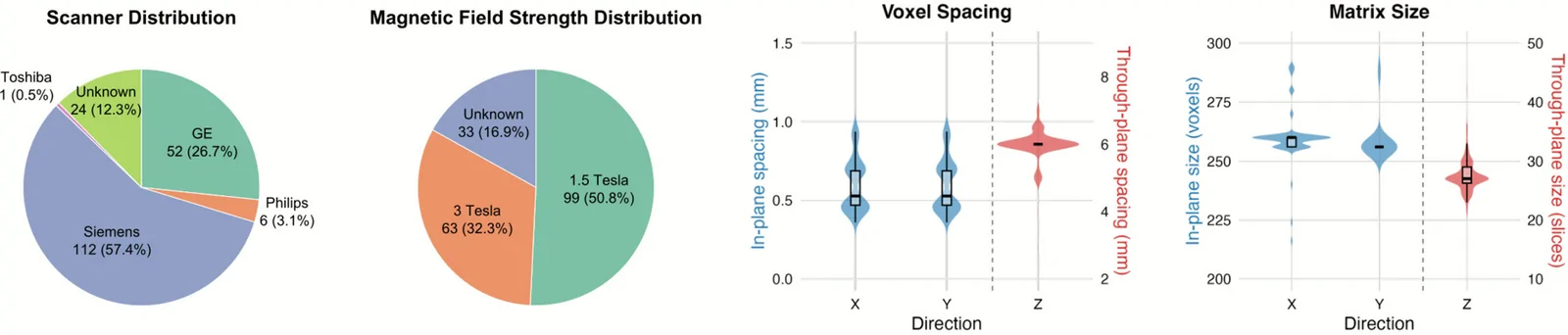
Overview of acquisition parameters in the curated clinical MRI dataset, illustrating substantial heterogeneity in scanner vendor distribution, magnetic field strengths, and spatial resolution. Median values (central lines) and interquartile ranges (boxes) are shown for voxel spacing and image matrix dimensions

A total of 747 routine clinical brain MRI scans from adults (≥ 18 years) evaluated in the Emory Cognitive Neurology Clinic between June 2006 and October 2022 were retrospectively extracted from the Goizueta Institute Data Platform (IRB: STUDY00005442) and analyzed under a secondary-use protocol (IRB: STUDY00006057). All participants consented to share medical records, including imaging and cerebrospinal fluid biomarkers, for research on cognitive disorders. Of these, 586 scans included both axial T2-weighted fluid-attenuated inversion recovery (FLAIR) and T1-weighted sequences. From this set, 195 scans from unique participants were consecutively selected for expert WMH annotation (Fig. 1). The number of annotated scans was determined by balancing dataset size with the substantial time and effort required for accurate manual labeling, while ensuring sufficient diversity for model training. To verify that the annotated subset was representative, we compared the distributions of scanner vendor, field strength, and image resolution between the subset and the full dataset. No significant differences were observed across these variables (Supplementary Table 1). The cohort included 101 males and 94 females (median age, 70 years [IQR, 63–74 years]; range 46–87 years). MRI was acquired on 71 scanners from four vendors (Siemens, GE, Philips, and Toshiba) at 1.5 or 3 Tesla (Fig. 2). Vendor and field strength information was occasionally missing due to de-identification or PACS export limitations, and is reported as “unknown.” All metadata and pixel-level personal health information (PHI) were removed in accordance with HIPAA Safe Harbor standards and the General Data Protection Regulation (GDPR), with metadata fields either deleted or replaced with de-identified values and any PHI in image pixels irreversibly obscured. The dataset exhibited substantial heterogeneity in voxel spacings and matrix sizes, reflecting real-world clinical variability (Fig. 2).

Of the 195 scans, 146 were used for training and 49 for testing, with the test set comprising 23 GE and 26 Siemens scans. Philips and Toshiba scanners contributed fewer than 4% of the annotated dataset, and their limited representation was insufficient to support generalizable test performance estimates. For this reason, the test set was limited to Siemens and GE, while scans from all four vendors were retained during model training. All images were manually annotated for WMH using ITK-SNAP 4.2 (https://www.itksnap.org) by an experienced imaging scientist (J.W.), following the STRIVE (STandards for ReportIng Vascular changes on Neuroimaging) criteria. Borderline or faint periventricular hyperintensities were labeled as WMH only when they were visually hyperintense relative to cortical gray matter and contiguous with white-matter regions; punctate signal changes suspected to reflect noise, motion, or partial-volume effects were excluded. Annotations were subsequently reviewed and refined by a fourth-year radiology trainee (J.D.B.) under the supervision of a board-certified neuroradiologist (R.H.), and all segmentations were iteratively corrected to consensus, providing a consistent, expert-validated reference standard for model training and evaluation.

### Preprocessing

N4 bias field correction was applied to both T1-weighted and FLAIR images using ANTs 2.2 (https://github.com/ANTsX/ANTs) to reduce low-frequency intensity non-uniformities. The bias-corrected T1-weighted image was then rigidly co-registered to the corresponding FLAIR image using FLIRT from FSL 6.0 (https://fsl.fmrib.ox.ac.uk). All T1-to-FLAIR registrations underwent visual quality control, with no gross misalignment detected. Image quality was also reviewed to confirm that motion or other artifacts did not compromise downstream processing.

### nnU-Net Model Training

A U-Net-based model was developed using the nnU-Net framework [10], which automatically configures the network architecture, preprocessing, data augmentation, and training hyperparameters based on the characteristics of the input dataset. Both training and inference were conducted using FLAIR and co-registered T1-weighted images. The model was trained using nnU-Net’s default settings, including stochastic gradient descent with Nesterov momentum (*μ* = 0.99) as the optimizer, an initial learning rate of 1 × 10^−3^ decayed using a polynomial schedule (power = 0.9), a batch size of 2, and a loss function composed of the unweighted sum of Dice loss and cross-entropy loss. Training was performed using five-fold cross-validation on 146 training scans. The five resulting models were combined into an ensemble by averaging voxel-wise softmax probabilities, which were then applied to the 49 test scans for final evaluation. This ensemble model is hereafter referred to as Robust‑WMH‑UNet.

### Baseline Comparison

To benchmark segmentation performance, the nnU-Net model was evaluated against several established and recently developed WMH segmentation methods, including the LST Lesion Prediction Algorithm (LPA), LST-AI [6], WMH-SynthSeg [7], and MindGlide [12], as well as the leading methods from the WMH Segmentation Challenge 2017: pgs [13] and sysu_media [14]. LST LPA is a classical model–based algorithm that utilizes a logistic regression framework to generate lesion probability maps. WMH-SynthSeg and MindGlide employ synthetic data and domain randomization techniques to enhance robustness across diverse imaging protocols. LST-AI, pgs, and sysu_media are U-Net-based deep learning methods incorporating ensemble learning strategies.

Inference was conducted using pretrained models for LST LPA, LST-AI, WMH-SynthSeg, MindGlide, pgs, and sysu_media. Specifically, LST LPA, WMH-SynthSeg, and MindGlide operated on FLAIR images exclusively, while LST-AI, pgs, and sysu_media required both FLAIR and co-registered T1-weighted images as inputs.

Segmentation performance was quantitatively assessed against the expert manual annotations using the Dice similarity coefficient (DSC), false-positive rate (FPR), and false-negative rate (FNR). Group comparisons with nnU-Net were performed using the Wilcoxon signed-rank test, with false discovery rate (FDR) adjustment for multiple comparisons. Statistical analysis was conducted in R 4.4 (R Foundation for Statistical Computing, Vienna, Austria), with significance defined as FDR-corrected *P* < 0.05. To examine sensitivity for small lesions, lesions were stratified by size into small (< 50 mm^3^), medium (50–500 mm^3^), and large (> 500 mm^3^). Lesion-wise detection rates were calculated using a 10% overlap fraction relative to ground truth and pooled across all lesions in the test set.

### MedSAM Model Fine-Tuning

To assess the utility of foundation models for clinical WMH segmentation, we fine-tuned MedSAM, a Segment Anything Model (SAM) [15] pretrained on diverse medical imaging tasks [11]. Since MedSAM operates on 2D inputs, axial slices were extracted from both FLAIR and co-registered T1-weighted images. Each three-channel input was constructed by duplicating the FLAIR slice across two channels and incorporating the corresponding T1-weighted slice as the third channel. To facilitate whole-brain segmentation without relying on manually defined prompts, the bounding box input was set to cover the whole image. Fine-tuning was performed on the training set using the AdamW optimizer (*β*_1_ = 0.9, *β*_2_ = 0.999) with an initial learning rate of 1 × 10^−4^, weight decay of 0.1, and a batch size of 4, over 30 epochs. The loss function was defined as the unweighted sum of Dice loss and cross-entropy loss. During training, the prompt encoder was frozen, while the image encoder and mask decoder were updated. Model performance was evaluated on the 49 held-out test scans from our clinical dataset. The resulting model is hereafter referred to as Robust-WMH-SAM.

## Results

### Cohort and Imaging Characteristics

The training set comprised 146 participants (median age, 69 years [IQR, 62–74 years]; range, 48–87 years; 73 females), including 88 with Alzheimer’s disease (AD) (60.3%), 9 with mild cognitive impairment (MCI) (6.2%), 46 cognitively normal (31.5%), and 3 with unknown diagnosis (2.1%). The test set included 49 participants (median age, 73 years [IQR, 66–77 years]; range, 46–84 years; 21 females), with 27 AD (55.1%), 4 MCI (8.2%), 17 cognitively normal (34.7%), and 1 with unknown diagnosis (2.0%).

The curated clinical dataset exhibited substantial heterogeneity in acquisition parameters, including scanner vendor, field strength, and spatial resolution (Fig. 2). Among the 195 MRI scans, Siemens (*N* = 112, 57.4%) and GE (*N* = 52, 26.7%) accounted for the majority of cases. Approximately half of the scans were acquired at 1.5 Tesla. The voxel spacing of FLAIR images varied considerably across the dataset, with in-plane spacing (*X* and *Y*) ranging from 0.36 mm to 0.94 mm, and through-plane spacing (*Z*) ranging from 3 to 7 mm. The image matrix dimensions also showed variation, ranging from 216 to 520 (*X*), 256 to 640 (*Y*), and 21 to 52 slices (*Z*).

**Fig. 3:**
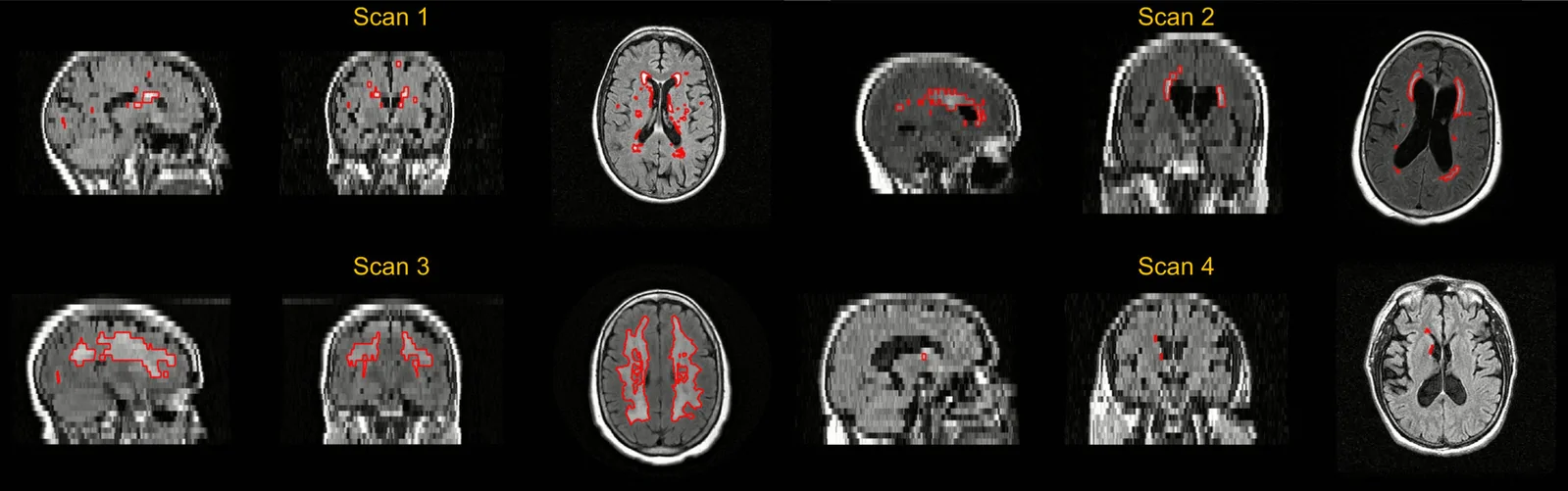
Representative FLAIR images with manual WMH annotations (red contours), illustrating variability in noise level, contrast, spatial resolution, lesion size, and shape

Figure 3 shows representative annotated FLAIR images, illustrating the presence of noise, limited spatial resolution, and substantial variability in lesion appearance, size, and contrast.

### Performance of the nnU-Net Model

**Fig. 4:**
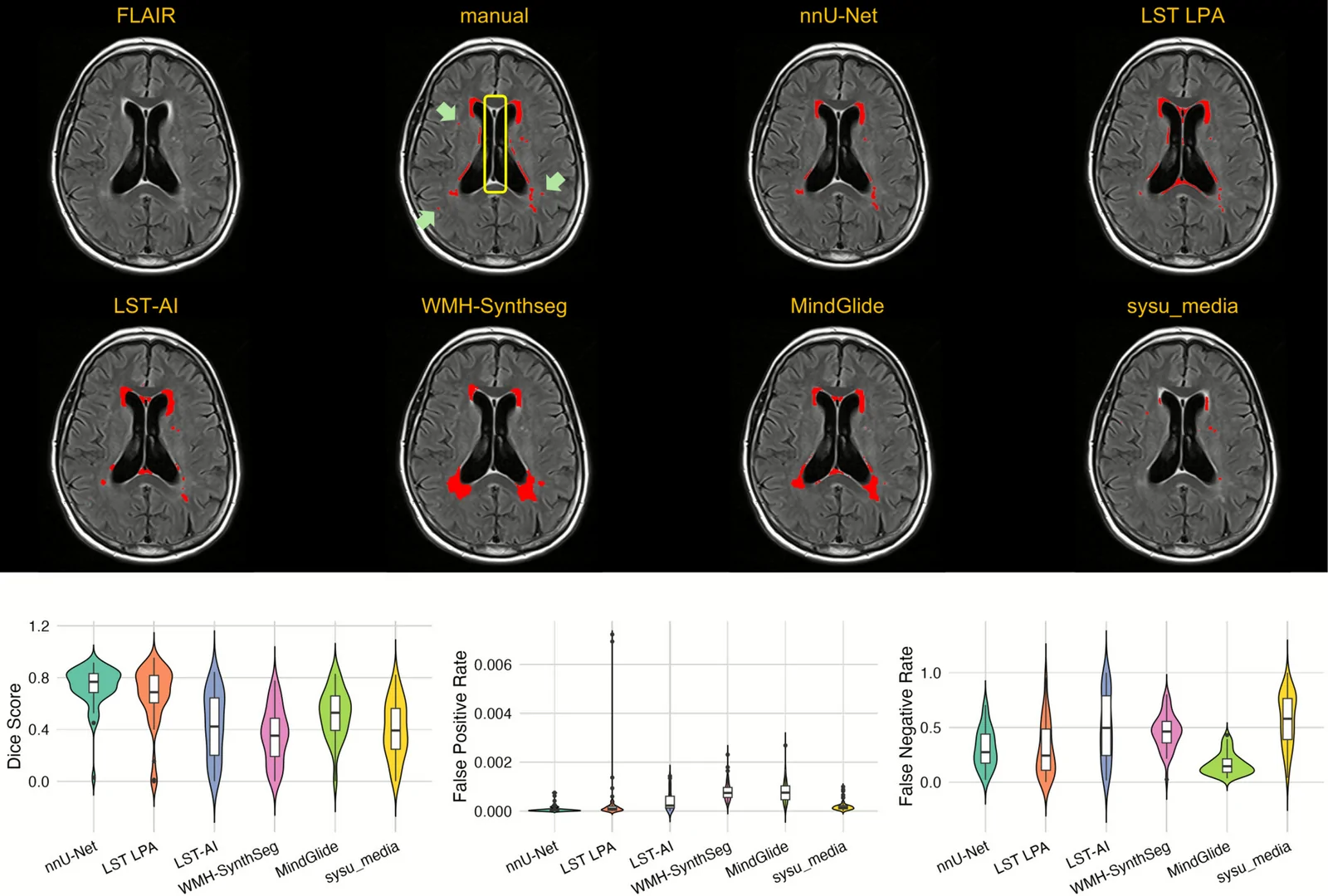
Example WMH segmentation results from different methods (upper panel). Smaller lesions (green arrows) were frequently missed, and LST LPA, LST-AI, and MindGlide misclassified the septum pellucidum (yellow rectangle) as WMH. WMH-SynthSeg and MindGlide showed extensive false positives, whereas sysu_media captured only a limited portion of WMHs. Quantitative performance on the test set, with median values (central lines) and interquartile range (boxes) for Dice similarity coefficient, false-positive rate, and false-negative rate (lower panel). Robust-WMH-UNet achieved the highest Dice score and lowest false-positive rate; MindGlide showed the lowest false-negative rate but relatively high false-positive rate

Example segmentation outputs from different WMH segmentation methods are shown in Fig. 4. pgs was excluded from evaluation due to a matrix size limit (≤ 500 in *X* or *Y*), which was exceeded in our dataset (up to 520 × 640), resulting in successful segmentation for only 13 of 49 cases (26.5%). While the remaining methods were able to roughly detect WMH, they struggled to identify smaller lesions. LST LPA, LST-AI, and MindGlide incorrectly classified the septum pellucidum, the thin membrane separating the lateral ventricles, as a WMH. WMH-SynthSeg and MindGlide produced segmentation maps with relatively large areas of false positives, whereas sysu_media detected only a small portion of WMHs, resulting in a high FNR.

Among the evaluated methods, Robust-WMH-UNet (nnU-Net trained on our clinical dataset) achieved the highest DSC on the test set (median, 0.768 [IQR, 0.686–0.832]; 95% CI, 0.710–0.809), significantly outperforming LST-AI (0.423 [0.201–0.644]; 95% CI, 0.350–0.560; FDR-corrected *P* < 0.001), WMH-SynthSeg (0.352 [0.192–0.487]; 95% CI, 0.300–0.443; FDR-corrected *P* < 0.001), MindGlide (0.529 [0.393–0.659]; 95% CI, 0.444–0.593; FDR-corrected *P* < 0.001), and sysu_media (0.393 [0.248–0.563]; 95% CI, 0.295–0.499; FDR-corrected *P* < 0.001). Robust-WMH-UNet also demonstrated higher median DSC than LST LPA (0.688 [0.606–0.817]; 95% CI, 0.646–0.786), although this difference did not remain statistical significance after FDR correction (*P* = 0.143) (Fig. 4). In terms of error rates, Robust-WMH-UNet achieved the lowest FPR (3.870 × 10^−5^ [1.286 × 10^−5^–8.485 × 10^−5^]; 95% CI, 1.730 × 10^−5^–5.498 × 10^−5^) with a moderate FNR (0.273 [0.173–0.438]; 95% CI, 0.226–0.370). In contrast, MindGlide yielded lowest FNR (0.144 [0.090–0.211]; 95% CI, 0.103–0.159) but a relatively high FPR (7.552 × 10^−4^ [4.659 × 10^−4^–1.032 × 10^−3^]; 95% CI, 5.648 × 10^−4^–8.732 × 10^−4^), reflecting frequent misclassification of large regions as WMH.

To assess sensitivity for detecting small lesions, we performed a lesion-size-stratified analysis, which showed that Robust-WMH-UNet had a lower lesion-wise detection rate for small WMHs (< 50 mm^3^; 0.500 [95% CI, 0.465–0.535]) compared with medium (50–500 mm^3^; 0.815 [95% CI, 0.773–0.851]) and large (> 500 mm^3^; 0.991 [95% CI, 0.952–0.998]) lesions (Supplementary Table 2).

To further evaluate the robustness and generalizability of Robust-WMH-UNet, we tested the model on the public WMH Segmentation Challenge 2017 test set without retraining, using the challenge-provided annotations. The model achieved a median DSC of 0.724 ([IQR, 0.596–0.805]; 95% CI, 0.660–0.749), indicating reasonable out-of-sample performance on a standardized research dataset.

### Evaluation of the Foundation Model

**Fig. 5:**
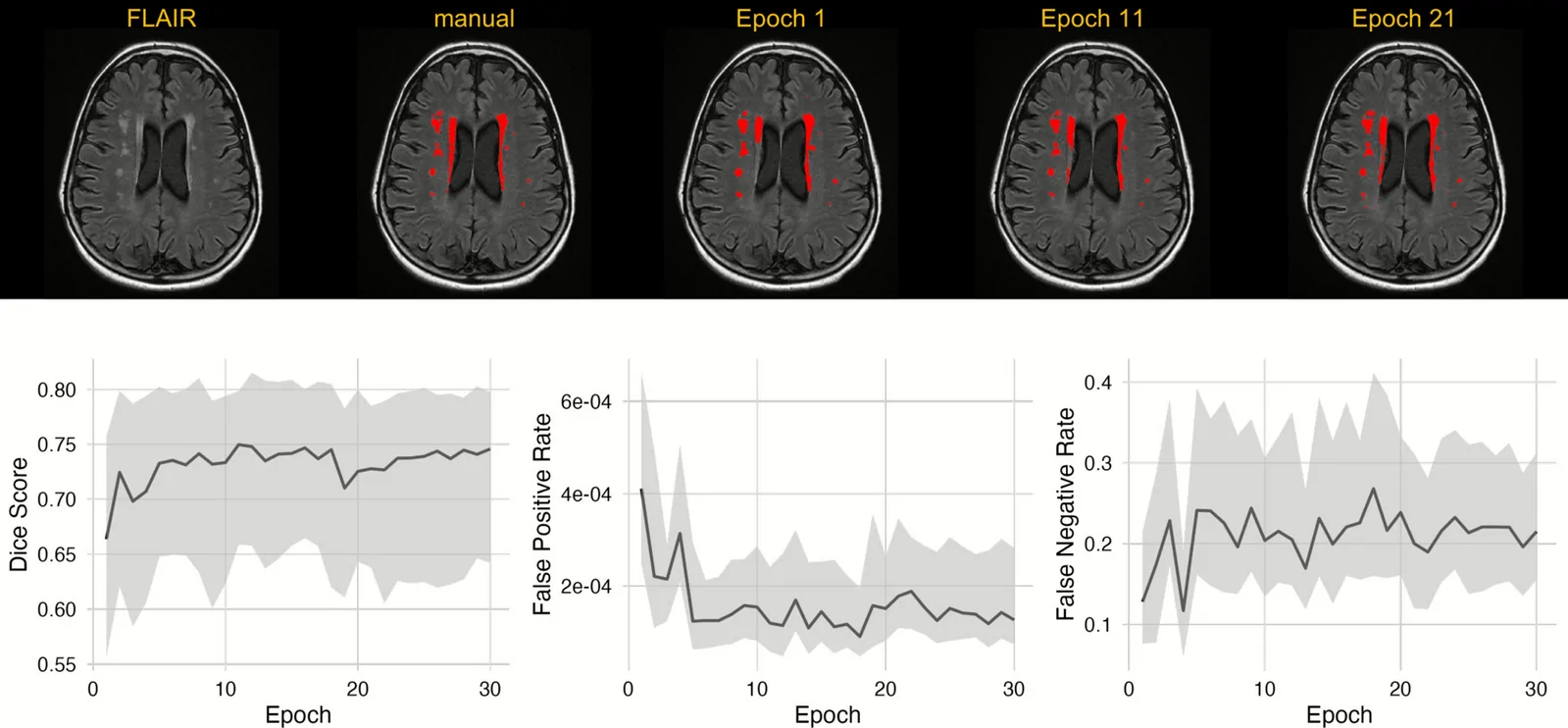
Representative segmentations by Robust-WMH-SAM at epochs 1, 11, and 21 (upper panel), illustrating progressive refinement and increased lesion coverage. Quantitative performance on the test set across fine-tuning epochs, with median values (solid lines) and interquartile ranges (shaded areas) for Dice similarity coefficient, false-positive rate, and false-negative rate (lower panel). Metrics stabilized after epoch 5, reaching a maximum at epoch 11

Robust-WMH-SAM, obtained by fine-tuning MedSAM on our clinical training set, demonstrated promising performance even with limited training epochs (Fig. 5). After just one epoch of fine-tuning, the model detected most WMH regions, with lesion delineations progressively expanding to more comprehensively encompass the white matter lesions by epochs 11 and 21.

Robust-WMH-SAM achieved a DSC of 0.664 [0.557–0.757] after fine-tuning MedSAM for a single epoch. The highest DSC was observed at 11 epochs (0.750 [0.659–0.798]), approaching the performance of nnU-Net. From epoch 5 onward, DSC, FPR, and FNR values remained relatively stable.

To evaluate whether the 2 × FLAIR + 1 × T1w input configuration was optimal, we trained two additional variants (1 × FLAIR + 2 × T1w and 3 × FLAIR) using the same protocol. The 2 × FLAIR + 1 × T1w configuration yielded the highest median DSC across epochs (0.737 [0.729–0.742]), whereas the 3 × FLAIR variant showed lower accuracy and greater variability (0.677 [0.648–0.716]) (Supplementary Fig. 1). These results support the selection of 2 × FLAIR + 1 × T1w as the primary input configuration for Robust-WMH-SAM.

A qualitative review identified five typical failure modes in WMH segmentation methods: (1) missed small punctate WMHs, (2) anatomical confounders (e.g., the septum pellucidum), (3) ambiguous lesion boundaries in periventricular or confluent regions, (4) erroneous WMH predictions located within gray matter, and (5) other hyperintense lesions that mimic WMH (e.g., post-radiation changes) (Supplementary Fig. 2). Missed small lesions and confounder-induced false positives, including post-radiation effects, were observed across all segmentation models, with unreliable boundary delineation being particularly common in scans with low lesion-to-background contrast. Robust-WMH-UNet and Robust-WMH-SAM produced more accurate delineations in several of these challenging cases.

## Discussion

This study presents three key contributions to WMH segmentation in heterogeneous clinical MR images: (1) the introduction of a diverse clinical WMH dataset, (2) the establishment of a robust nnU-Net baseline, and (3) the demonstration that a fine-tuned MedSAM can achieve strong performance with minimal training. This dataset, acquired across multiple vendors, field strengths, and acquisition protocols, provides a realistic benchmark for advancing the development and evaluation of WMH methods in real-world settings.

Our newly curated dataset addresses an important gap in WMH segmentation research by capturing the full diversity of routine clinical imaging. In contrast, widely used benchmark datasets, such as the WMH Segmentation Challenge 2017, were collected under controlled conditions, with standardized acquisition protocols, uniform field strengths, and consistent spatial resolutions. While these settings yield high-quality images with minimal artifacts and relatively homogeneous lesion characteristics, they do not adequately represent the variability inherent in clinical practice [9]. By encompassing a wider range of scanner vendors, field strengths, acquisition settings, image quality, and lesion burden, our dataset offers a more representative and challenging resource for developing robust segmentation methods.

Within this context, the strong performance of Robust-WMH-UNet underscores the importance of training on data that truly reflects the target clinical domain. Many established automatic WMH segmentation tools, despite achieving excellent results on standardized research datasets, showed marked performance drops when applied to our clinical MR images. This degradation is plausibly due to discrepancies in intensity distributions, spatial resolution, and the prevalence of imaging artifacts between research and routine images. In contrast, nnU-Net trained on our dataset consistently delivered high segmentation quality, revealing that supervised models trained on representative clinical data can substantially improve WMH delineation in real-world settings. These findings emphasize the need for training paradigms and benchmarking practices that prioritize clinical representativeness, thereby enhancing model generalizability and supporting reliable deployment in diverse healthcare environments.

In interpreting the cross-dataset results, note that the WMH Segmentation Challenge 2017 dataset employs a more conservative annotation protocol than our clinical dataset, which affects direct DSC comparisons. Moreover, published WMH17 leaderboard scores were obtained using models trained specifically on the WMH17 training set, whereas our evaluation was performed without any dataset-specific retraining. These differences likely account for the modest gap between our out-of-sample performance on the WMH17 test set (median DSC = 0.724) and the top challenge submissions (DSC ≈ 0.75–0.81). Notably, the leading WMH17 model (sysu_media), despite being optimized exclusively for that dataset, exhibited substantially lower performance on our heterogeneous clinical scans, underscoring the need for clinically representative training data to achieve robust, real-world WMH segmentation.

Building upon these insights, foundation models have emerged as a promising approach in medical image segmentation. Such models, pretrained on massive datasets and capable of adapting to new tasks, demonstrate remarkable generalizability [16]. For instance, MedSAM, a segment-anything transformer fine-tuned on over 1.5 million medical image-mask pairs, outperformed the original SAM and matched or surpassed specialist models on diverse segmentation tasks [11]. Although Robust-WMH-SAM did not outperform Robust-WMH-UNet on this WMH task, its notable accuracy achieved with limited fine-tuning demonstrates the potential of foundation models to generalize effectively in data-scarce or previously unseen clinical settings. This approach provides a scalable and efficient strategy for adapting large pretrained models to WMH segmentation, circumventing the need for computationally intensive training from scratch. Therefore, foundation models such as SAM may serve as valuable backbone initializations or pretraining frameworks, enhancing performance across a broad range of downstream clinical segmentation applications.

In examining error patterns, false negatives were most often associated with small WMH lesions, a limitation present even in the best-performing methods, Robust-WMH-UNet and Robust-WMH-SAM. This finding aligns with the WMH Segmentation Challenge 2017, which also reported higher false-negative rates for tiny lesions, underscoring the difficulty of detecting them [8]. False positives frequently arose in the septum pellucidum, the thin membrane separating the lateral ventricles, as it appears hyperintense on FLAIR much like WMHs [8]. Ambiguous lesion boundaries in periventricular or confluent zones, together with erroneous WMH predictions in gray matter, further contributed to segmentation errors. Robust-WMH-UNet and Robust-WMH-SAM consistently demonstrated improved delineation across these challenging scenarios. Although WMH-SynthSeg and MindGlide, both contrast-agnostic methods capable of accommodating heterogeneous clinical scans, performed well in whole-brain structure extraction [7, 12], their accuracy in WMH detection was lower than that of Robust-WMH-UNet and Robust-WMH-SAM.

Although Robust-WMH-UNet and Robust-WMH-SAM were developed using GPU-accelerated training, their inference requirements remain compatible with lower-resource environments. nnU-Net-based inference can technically run on CPU-only systems, though processing times increase substantially for large 3D volumes. In contrast, MedSAM-based inference (SAM ViT-B backbone) has modest computational demands and can be executed on standard desktop CPUs with 8–16 GB RAM. Importantly, both models do not require high-end GPUs for deployment, and their memory demands for inference are substantially lower than those typically needed for training. These capabilities support the feasibility of deploying our models in settings with limited computational resources.

This study has several limitations. First, the dataset included only axial acquisitions, which may restrict generalizability. Incorporating sagittal, coronal, and volumetric 3D data could further improve robustness. Second, primary WMH annotations were produced by a single rater and subsequently reviewed to expert consensus. Although this process reduces subjective variability, the absence of quantitative inter-rater reliability statistics may limit the robustness of the reference standard. Third, the deep learning models used only FLAIR and T1-weighted sequences, and domain randomization strategies, e.g., SynthSeg [17], may enhance adaptability to varying image contrasts. Fourth, the lower detection rate for small WMHs reflects an inherent limitation of current WMH segmentation methods. Future work may explore lesion-aware loss functions or multi-scale architectures to improve sensitivity for small-lesion detection. Fifth, the foundation model was implemented in 2D, limiting volumetric context capture. Extending SAM to a fully 3D framework is a key direction for future work. Finally, this study was retrospective; a prospective clinical validation is required to establish practical utility. Such a study should evaluate model performance on consecutively acquired routine scans, incorporate fresh blinded manual annotations as reference standards, and assess workflow integration including failure modes and processing time.

In summary, we introduce a diverse clinical MRI dataset with expert-annotated WMHs that reflects the inherent variability of real-world imaging. We expect the dataset to accelerate the development of robust WMH algorithms, enable fair cross-method comparisons in clinically relevant scenarios, and facilitate the translation of segmentation models into routine care. Models trained on this dataset demonstrate clear performance gains over established benchmark methods, highlighting the importance of clinically representative training data. Our dual approach, i.e., combining the development of a robust, task-specific model with the fine-tuning of a foundation model, provides scalable and generalizable solutions for WMH segmentation in routine neuroimaging practice.

## Supplementary Information

Below is the link to the electronic supplementary material.

ESM 1 (PDF 406 KB)

## Data Availability

The Emory WMH dataset, described and curated in this study, is available at https://doi.org/10.5281/zenodo.18339994.
