# Supplementary material for: Benchmark White Matter Hyperintensity Segmentation Methods Fail on Heterogeneous Clinical MRI: A New Dataset and Deep Learning–Based Solutions
Source: J Imaging Inform Med. 2026 Jan 29;39(5):4463–71. doi: 10.1007/s10278-025-01808-9 (PMC13694494; doi:10.1007/s10278-025-01808-9)
Supplement: Supplementary file 1 [file 10278_2025_1808_MOESM1_ESM.pdf]

Benchmark white matter hyperintensity segmentation methods fail on heterogeneous clinical MRI: A new dataset and deep learning-based solutions

Supplementary Table 1. Comparison of acquisition parameters between the annotated subset and the full MRI dataset

	Category	Annotated subset (*N* = 195)	Full dataset (*N* = 586)	Test	Uncorrected *P*	FDR-corrected *P*[1]
Scanner				Fisher exact	.237	.377
	GE	26.7%	22.7%			
	Philips	3.1%	3.2%			
	Siemens	57.4%	55.6%			
	Toshiba	0.5%	0.2%			
	Unknown	12.3%	18.3%			
Magnetic field strength				Fisher exact	.386	.377
	1.5 Tesla	50.8%	54.3%			
	3 Tesla	32.3%	27.1%			
	Unknown	16.9%	18.6%			
		Median [IQR]	Median [IQR]			
Voxel spacing X (mm)		0.527 [0.469 - 0.688]	0.488 [0.449 - 0.688]	KS-perm	.105	.365
Voxel spacing Y (mm)		0.527 [0.469 - 0.688]	0.488 [0.449 - 0.688]	KS-perm	.183	.365
Voxel spacing Z (mm)		6.000 [6.000 - 6.000]	6.000 [6.000 - 6.000]	KS-perm	.283	.377
Matrix size X		384 [260 - 512]	384 [260 - 512]	KS-perm	.181	.365
Matrix size Y		512 [320 - 512]	512 [320 - 512]	KS-perm	.136	.365
Matrix size Z		27 [26.5 - 29]	27 [26 - 29]	KS-perm	.640	.640

Differences in categorical distributions (i.e., scanner vendor and magnetic field strength) were assessed using Fisher's exact test. Differences in continuous variable distributions (i.e., voxel spacing and matrix size) were evaluated using a permutation-based Kolmogorov-Smirnov test (KS-perm; 10,000 permutations).
[1]*P* values were adjusted for multiple comparisons using the false discovery rate (FDR) correction.

Supplementary Table 2. Lesion-wise detection rates stratified by lesion size

	Small (< 50 mm^3)	Medium (50 - 500 mm^3)	Large (> 500 mm^3)
nnUNet	0.500 [95% CI: 0.465 - 0.535]	0.815 [95% CI: 0.773 - 0.851]	0.991 [95% CI: 0.952 - 0.998]
LST LPA	0.446 [95% CI: 0.412 - 0.481]	0.707 [95% CI: 0.659 - 0.751]	0.991 [95% CI: 0.952 - 0.998]
LST-AI	0.538 [95% CI: 0.503 - 0.573]	0.718 [95% CI: 0.670 - 0.761]	0.842 [95% CI: 0.764 - 0.898]
WMH-SynthSeg	0.236 [95% CI: 0.207 - 0.267]	0.533 [95% CI: 0.483 - 0.583]	0.974 [95% CI: 0.925 - 0.991]
MindGlide	0.422 [95% CI: 0.388 - 0.457]	0.836 [95% CI: 0.796 - 0.870]	0.991 [95% CI: 0.952 - 0.998]
sysu_media	0.709 [95% CI: 0.676 - 0.740]	0.813 [95% CI: 0.770 - 0.849]	0.939 [95% CI: 0.879 - 0.970]

All results are pooled across all lesions in the test set.
Lesion-wise detection rates were determined using a 10% overlap fraction relative to ground truth.

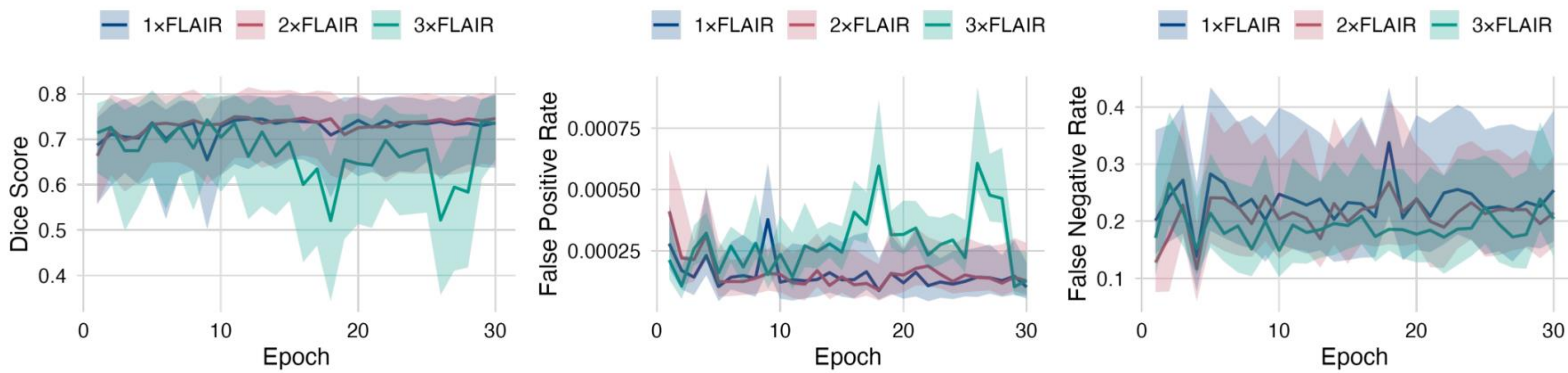

Supplementary Figure 1. Performance of Robust-WMH-SAM on the test set across fine-tuning epochs for three input configurations (1×FLAIR + 2×T1w, 2×FLAIR + 1×T1w, and 3×FLAIR). Solid lines represent median values and shaded regions indicate interquartile ranges for the Dice similarity coefficient (left), false positive rate (middle), and false negative rate (right). Among the three configurations, 2×FLAIR + 1×T1w achieved the highest median DSC across epochs, whereas 3×FLAIR showed reduced accuracy and greater variability.

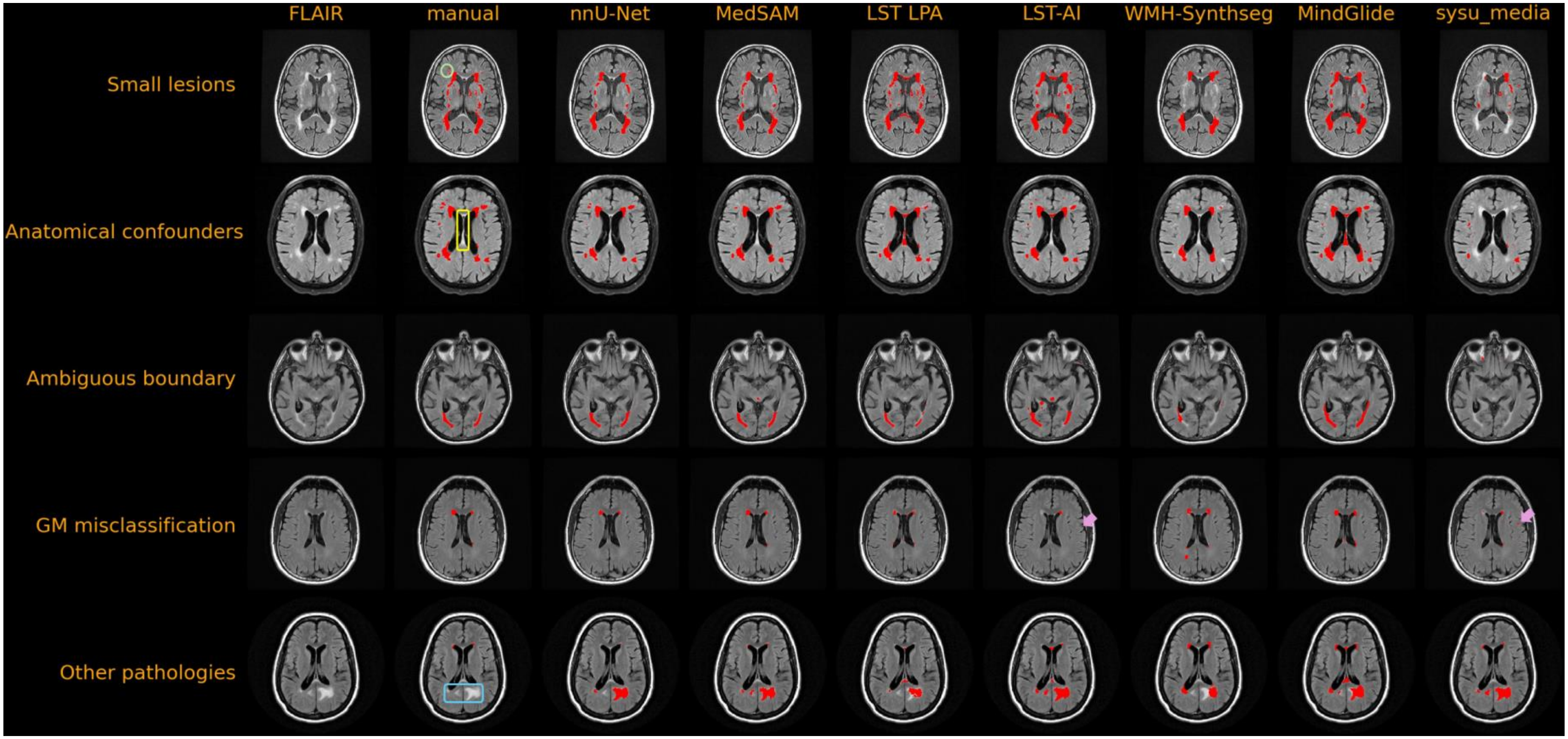

Supplementary Figure 2. Qualitative examples of five typical failure modes: (1) missed small punctate WMH lesions (green circle), (2) anatomical confounders such as the septum pellucidum (yellow rectangle), (3) ambiguous periventricular or confluent lesion boundaries, (4) erroneous WMH predictions located within gray matter (GM) (purple arrows), and (5) other hyperintense pathologies that mimic WMH, such as post-radiation changes (blue rectangle). Missed small lesions and confounder-induced false positives, including post-radiation effects, are observable across all WMH segmentation models. Robust-WMH-UNet and Robust-WMH-SAM demonstrate improved delineation in several of these challenging scenarios.
